# The effect of secondhand smoke exposure on dental caries and gingival health among schoolchildren in Damascus, Syria: a cross-sectional study

**DOI:** 10.1186/s12903-023-03486-x

**Published:** 2023-10-11

**Authors:** Ghalia Misrabi, Mawia Karkoutly, Nada Bshara

**Affiliations:** Pediatric Dentistry Department, Faculty of Dentistry, Damascus University, Damascus, Syria

**Keywords:** Secondhand smoke, Cigarette tar, Dental caries, Dental plaque, Oral health

## Abstract

**Objective:**

This study aimed to evaluate the effect of secondhand smoke on dental caries and gingival health among schoolchildren in Damascus, Syria.

**Materials and methods:**

This was a cross-sectional study. It was carried out at government schools in Damascus, Syria. This study included healthy children aged 10 to 13 years old. Schoolchildren were interviewed to answer the researcher-administered questionnaire to obtain answers regarding demographic information and family smoking behavior. A dental examination was performed by a dentist, and the number of decayed (D), missing (M), and filled (F) permanent teeth (DMFT) was scored based on the World Health Organization (WHO) 1997. A gingival examination was performed using the modified gingival index (MGI) and Silness-Leo plaque index (PI) to assess gingival inflammation and plaque accumulation, respectively.

**Results:**

A total of 284 schoolchildren participated. More than half (61.26%) of them were exposed to secondhand smoke (SHS), and about half of them (52.11%) resided in a house with at least one cigarette smoked in a day. About one-third of the passive smokers (33.30%) had poor plaque control, with a statistically significant difference from non-passive smokers (p < 0.05). The multivariate regression model showed that the number of smokers at home was significantly associated with the DMFT score, dental plaque accumulation, and gingival inflammation (p < 0.1). However, the number of cigarettes smoked at home in a day was not a predictor for dental caries and gingival status (p = 1.000).

**Conclusions:**

Within the limitations of this study, the number of smokers at home appears to have more adverse effects on children’s oral health compared to the quantity of smoke inhaled. In addition, SHSe was associated with more dental plaque accumulation among schoolchildren.

## Introduction

Smoking habits are usually acquired as a mimicry of the surroundings or to relieve stress during certain hardships [1]. According to Kakaje et al., the war-induced stressors during the Syrian crisis increased tobacco use [2]. Smoking can cause medical and oral conditions such as cardiovascular disease, stroke, cancer, lung conditions, chronic obstructive pulmonary disease, and periodontal diseases [3, 4]. However, passive smokers are prone to the same dangerous conditions as smokers because of secondhand smoke exposure (SHSe). SHSe is the inhalation of smoke exhaled by individuals who smoke or smoke from different tobacco products, such as cigarettes or hookahs [5]. Since 1964, it is believed that about 3 million passive smokers died due to the health conditions caused by SHSe [6]. Unfortunately, around 40–50% of the world’s children are passive smokers, and children of nicotine-dependent parents are more likely to smoke themselves [7]. Moreover, almost 80% of smokers reside in middle- and low-income countries [2]. This addresses the need to shed light on parents’ smoking behavior. Since the 1990s, hookah smoking has been considered a pleasurable yet epidemiological trend since it has many harmful effects on the environment and society [8]. Unfortunately, smoking hookah in Syria is considered a cultural practice and increases the sense of togetherness in daily socializing [2]. However, hookah smoke is as harmful as smoking cigarettes, and about 12% of children who are exposed to hookah smoke are more likely to report nasal congestion and wheezing [9].

Recent evidence has confirmed the association between SHSe and oral conditions. SHSe can adversely affect the immunity responses by decreasing serum immunoglobulin G (IgG) and saliva immunoglobulin A (IgA) levels, resulting in periodontitis and tooth loss. In addition, SHSe reduces vitamin C levels and salivary flow, causing Streptococcus mutans and Lactobacillus proliferation, which results in dental caries [10]. Remarkably, dental caries and poor gingival health harm health-related quality of life (HRQoL) [11]. However, research findings are controversial since some studies have not confirmed the association between SHSe and oral diseases [12, 13]. Therefore, this study aimed to evaluate the effect of secondhand smoke on dental caries and gingival health among schoolchildren in Damascus, Syria. The null hypothesis is that no significant difference would be noted between passive smokers and non-passive smokers in dental caries, plaque accumulation, and gingival inflammation.

## Materials and methods

### Participants selection

This was a cross-sectional study. It was carried out at government schools in Damascus, Syria. Schools were representative of all geographic locations within Damascus. This study was carried out from January 2023 to March 2023. This study included healthy children aged 10 to 13 years old. Written informed consent was obtained from participants’ legal guardians. Children with systemic conditions and learning disabilities or children with fixed orthodontic appliances were excluded. The sample size was estimated using one proportion formula according to population proportion (p) 79.1%, based on a margin of error (e) of 5% and confidence level of (α) 95%. The sample size was calculated according to the caries prevalence of 79.1% in Damascene schoolchildren [14]. The sample size obtained was 255. Ethics approval was provided by the ethics committee for research at Damascus University (N 419/2023) then participants were recruited for the study. The participation of schoolchildren was voluntary.

### Questionnaire instrument

An online Arabic questionnaire was designed using Google Forms. It was created based on previously validated questionnaires [15–17]. In addition, Cronbach’s alpha reliability test was used to measure internal consistency, which adds to the questionnaire’s validity. Cronbach’s alpha reliability test has shown good values (0.9 > α ≥ 0.8). Because of its accurate screening, an interviewer-administered questionnaire was used, and respondents gained a deeper interpretation of survey questions if any further clarifications were required [18]. Schoolchildren were interviewed to answer the researcher-administered questionnaire to obtain answers regarding demographic information and family smoking behavior. The first section of the questionnaire collected demographic information, including sex (female; male), age (in years), and educational level of parents (no formal education, primary, secondary, or tertiary education). The second section collected data regarding SHSe and the toothbrushing behavior of the schoolchildren. Schoolchildren were asked about the number of smokers at home (zero, one, two, three, or more) and the number of cigarettes smoked inside the house in a day (zero, one, or more). In addition, there were asked about the father, mother, and other family members’ smoking status (smoker, non-smoker) and type (cigarettes, hookah, dual use). Furthermore, schoolchildren were asked about the frequency of brushing their teeth in a day (zero, one, two, three).

### Clinical examination

A dental examination was performed by a dentist using a dental mirror and an explorer after air drying and under natural light. The number of decayed (D), missing (M), and filled (F) permanent teeth were scored based on the World Health Organization (WHO) 1997 [19], and the DMFT index score was recorded. The gingival examination was performed using a Williams periodontal probe, modified gingival index (MGI) [20], and Silness-Leo plaque index (PI) [21] were used to assess gingival inflammation and plaque accumulation, respectively. The Kappa coefficient of intra-examiner reliability was > 0.8.

To assess the severity of gingivitis, the qualitative changes of the gingival tissues around the six index teeth for permanent dentition (16, 11, 24, 36, 41, and 44) [22] and mixed dentition (16, 11, 64, 36, 41, and 84) [23] were examined then the mean score was calculated for each patient. MGI was scored as follows:

0 = Normal gingiva.

1 = slight change in color and texture and no bleeding on probing.

2 = redness, edema, hypertrophy, and bleeding on probing.

3 = redness, hypertrophy, ulceration, and spontaneous bleeding.

To determine the dental plaque accumulation of the schoolchildren, a Williams periodontal probe was used to screen plaque on the four surfaces of the six index teeth for permanent dentition (16, 11, 24, 36, 41, and 44) [22] and mixed dentition (16, 11, 64, 36, 41, and 84) [23] then the mean score was calculated for each patient. Silness-Leo PI was scored as follows:

0 = No plaque.

1 = A thin film of plaque along the free gingival margin. It can be detected only by using a periodontal probe.

2 = Moderate plaque accumulation along the free gingival margin, and easier to detect.

3 = Severe plaque accumulation within the interproximal region and along the free gingival margin.

### Statistical analysis

Statistical analysis was performed using IBM SPSS Statistics 26 (IBM SPSS Statistic, Inc., Chicago, IL, USA). Descriptive statistics were performed and presented as means and standard deviation for continuous variables. In addition, it was presented as frequencies and percentages for categorical variables. Chi-square test was used to compare dental caries and gingival status among schoolchildren according to their smoking status, and the statistical significance level was set at 0.05 (p < 0.05). A multivariate regression model was performed to evaluate the relationship between independent variables (number of smokers at home and number of cigarettes smoked inside the house in a day) and DMFT, PI, and MGI as parameters. The statistical significance level was adjusted at 0.1 (p < 0.1).

## Results

A total of 284 schoolchildren participated in the current study. More than half (52.46%) of them were boys, and 43.66% of the participants were aged 11 years old. Approximately one-third (31.69%) of the schoolchildren’s fathers had completed their tertiary education, and more than one-third (36.97%) of the mothers had completed primary education (Table 1).

**Table 1:** Sociodemographic characteristics of the schoolchildren and their parents

Variables	n (%)
**Sex**	
Female Male	135 (47.54) 149 (52.46)
**Age**	
10 11 12 13	39 (13.73) 124 (43.66) 105 (36.97) 16 (5.63)
**Father’s educational level**	
No formal education Primary education Secondary education Tertiary education	65 (22.89) 86 (30.28) 43 (15.14) 90 (31.69)
**Mother’s educational level**	
No formal education Primary education Secondary education Tertiary education	43 (15.14) 104 (36.62) 64 (22.54) 73 (25.70)

More than half (61.26%) of the schoolchildren were exposed to secondhand smoke (SHS), and about half of them (52.11%) resided in a house with at least one cigarette smoked in a day. More than half (55.99%) of the schoolchildren’s fathers were smokers, with cigarettes being the most consumed (60.38%). The majority of the mothers (76.41%) were non-smokers. However, among smoking mothers, hookah was the most commonly smoked tobacco form (53.73%). Regarding toothbrushing behavior, about one-third of the schoolchildren (30.38%) reported brushing their teeth twice a day (Table 2).

**Table 2:** Secondhand smoke exposure, and toothbrushing behavior of the schoolchildren

Variables	n (%)
**Number of smokers at home**	
0 1 2 ≥ 3	110 (38.73) 87 (30.63) 62 (21.83) 25 (8.80)
**Number of cigarettes smoked inside the house in a day**	
0 ≥ 1	148 (52.11) 136 (47.89)
**Does your father smoke?**	
Yes No	159 (55.99) 125 (44.01)
**Father’s smoking type**	159 (100)
Cigarettes Hookahs Cigarettes and Hookahs	96 (60.38) 36 (22.64) 27 (16.98)
**Does your mother smoke?**	
Yes No	67 (23.59) 217 (76.41)
**Mother’s smoking type**	67 (100)
Cigarettes Hookahs Cigarettes and hookahs	22 (32.84) 36 (53.73) 9 (13.43)
**Does any other family member smoke?**	
Yes No	217 (76.40) 67 (23.59)
**Other family members’ smoking type**	38 (100)
Cigarettes Hookahs Cigarettes and hookahs	9 (23.68) 18 (47.37) 11 (28.95)
**How many times a day do you brush your teeth?**	
0 1 2 3	42 (14.79) 79 (30.28) 86 (30.38) 77 (27.11)

The vast majority of schoolchildren (90.49%) had at least one decayed, missing, or filled permanent tooth, and the mean ± standard deviation (SD) of DMFT score was 0.90 ± 0.29. In addition, more than half of them (57.39%) had fair dental plaque control, with a mean ± SD of 1.43 ± 0.62. Furthermore, the mean ± SD of gingival inflammation was 1.36 ± 0.54 (Table 3). There was no statistically significant difference between children of smokers and non-smokers in the DMFT score (p = 0.212) and gingival inflammation (p = 0.088). However, about a third of the passive smokers (33.30%) had poor plaque control, with a statistically significant difference from non-passive smokers (p < 0.05) (Table 4).

**Table 3:** DMFT score and gingival status of the schoolchildren

Variables	n (%)	Mean ± SD
**Number of decayed, missing, and filled teeth in the permanent dentition (DMFT score)**		0.90 ± 0.29
0 ≥ 1	27 (9.51) 257 (90.49)	
**Plaque index (PI) score**		1.43 ± 0.62
Excellent (0) Good (0.1–0.9) Fair (1.0-1.9) Poor (2.0–3.0)	1 (0.35) 53 (18.66) 163 (57.39) 67 (23.59)	
**Modified gingival index (MGI) score**		1.36 ± 0.54
0 0.1-1 1.1-2.0 2.1-3.0	9 (3.17) 44 (15.49) 227 (79.93) 4 (1.41)	

**Table 4:** Comparing dental caries and gingival status among schoolchildren according to their parents smoking status

Variables	Non-passive-smokers n (%)	Passive smoker n (%)	p-value
**Number of decayed, missing, and filled teeth in the permanent dentition (DMFT score)**	110 (100)	174 (100)	0.212
0 ≥ 1	7 (6.40) 103 (93.60)	20 (11.50) 154 (88.50)	
**Plaque index (PI) score**			< 0.05*
Excellent (0) Good (0.1–0.9) Fair (1.0-1.9) Poor (2.0–3.0)	1 (0.90) 41 (37.30) 59 (53.60) 9 (8.20)	0 (0.00) 12 (6.90) 104 (59.80) 58 (33.30)	
**Modified gingival index (MGI) score**			0.088
0 0.1-1 1.1-2.0 2.1-3.0	1 (0.90) 12 (10.90) 95 (86.40) 2 (1.80)	8 (4.60) 32 (18.40) 132 (75.90) 2 (1.10)	

*p < 0.05 = significant difference using Chi-square test; p values written in bold are statistically significant (p < 0.05)

The multivariate regression model showed that the number of smokers at home was significantly associated with the DMFT score, dental plaque accumulation, and gingival inflammation (p < 0.1). However, the number of cigarettes smoked at home in a day was not a predictor for dental caries and gingival status (p = 1.000) (Table 5).

**Table 5:** The multivariate regression model of DMFT, PI and MGI score

Independent variables	DMFT	PI	MGI
	p-value		
**Number of smokers at home**	< 0.1*		
Number of smokers at home 0 1 2 ≥ 3			
**Number of cigarettes smoked inside the house in a day**	1.000		
0 ≥ 1			

*p < 0.1 = significant difference; p values written in bold are statistically significant (p < 0.1)

## Discussion

Given the current prevalence of smoking and dental caries during the Syrian crisis, determining the oral conditions related to SHSe is a priority [2, 14]. In the current study, the authors hypothesized that exposure to SHS is a risk factor for dental plaque accumulation, dental caries, and gingival inflammation among children. Literature findings are controversial since some studies have not validated the correlation between SHSe and oral health conditions [12, 13]. To the best of the authors’ knowledge, this is the first study to shed light on the association between SHSe and dental caries and gingival health among schoolchildren in Damascus, Syria. Such research highlights the necessity of raising parental awareness regarding the harmful effect of SHSe on children’s oral health.

The results of the current study showed that there is a significant difference between passive smokers and non-passive smokers in terms of dental plaque accumulation. This result is in agreement with the one reported in Iran by Mosharrafian et al. [17], who suggested that passive smoking led to an increase in dental plaque accumulation. Nevertheless, this finding is in contrast to the one reported in Turkey [24]. This could be explained by the same mechanism of active smoking, which causes bacterial dental plaque build-up. Some chemicals in tobacco, such as nicotine and tar, reduce the salivary flow, which in turn causes the oral bacteria to stick to teeth and the surrounding tissues [25]. According to Sakki et al. [26], SHSe increases Streptococcus mutans and Lactobacillus levels. In addition, nicotine increases extracellular polysaccharides, which play an essential role in cohering microorganisms to dental plaque. A further explanation for this finding is that parents who smoke might be less likely to brush their teeth, and children may imitate their parents’ behavior [27]. However, in the current study, there was no statistically significant difference between passive smokers and non-passive smokers in the DMFT score. This could be explained by the fact that, permanent teeth in the current age group are newly erupted, and dental caries develop over time. However, this finding is in contrast with the results reported in Saudi Arabia and Iran [16, 17].

The result of the current study showed that the number of smokers at home was significantly associated with the DMFT score, dental plaque accumulation, and gingival inflammation among children. Nevertheless, the number of cigarettes smoked at home in a day was not a predictor for dental caries and gingival status. In other words, the number of people with a smoking habit at home has more adverse effects on children’s oral health compared to the quantity of smoke inhaled. This could be explained by the well-known fact that smoking habits were associated with poor dietary patterns, irregular breakfast, and unhealthy lifestyles. Hence, schoolchildren who are passive smokers are influenced by their parental dietary habits and low oral health awareness [28, 29]. In addition, poor nutrient intake has a crucial impact on the gingival inflammatory reaction and dental caries incidence [30, 31]. As previously stated, smokers brush their teeth less likely, and children can easily imitate their parents’ bad habits [27]. In addition, according to Mattheus et al. [32], smoking parents have a higher number of microorganisms in the oral cavity, which in turn cause vertical transmission to their offspring. Smokers have a lower intake of dietary fiber, polyunsaturated fatty acids, folates, magnesium, and antioxidant vitamins such as vitamins C, A, and E. In addition, Smokers have a greater intake of saturated fat, fried foods, carbohydrates, coffee, and alcohol [28, 29]. The aforementioned dietary patterns all have a profound impact on dental caries and gingival inflammation [30, 31]. Another possible explanation regarding the effect of cigarette numbers is the possibility that children were not reporting the numbers accurately and did not know the length of time of exposure.

### Limitations

This study had limitations. First, this is a cross-sectional study design that is unable to establish causality or provide incidence. A cohort or longitudinal study is mandatory to demonstrate causality and measure incidence [33]. Second, many confounding factors may affect DMFT, plaque accumulation, and gingival inflammation. Third, it was only conducted in the government schools of Damascus. Hence, study findings should be generalized with caution. Fourth, this survey is subjected to social desirability bias since it relies on children’s reports of their parents’ smoking habits. Therefore, children will choose the most favorable answers [34]. Last, more accurate methods should be applied in future studies to measure SHSe, such as serum cotinine level.

## Conclusions

Within the limitations of this study, the number of smokers at home appears to have more adverse effect on children’s oral health compared to the quantity of smoke inhaled. In addition, SHSe was associated with more dental plaque accumulation among schoolchildren.

## Data Availability

The datasets generated during and/or analysed during the current study are available from the corresponding author on reasonable request.
